# Dogmatic modes of science

**DOI:** 10.1177/03010066211047826

**Published:** 2021-11-01

**Authors:** Roy S. Hessels, Ignace T. C. Hooge

**Affiliations:** Experimental Psychology, Helmholtz Institute, 8125 Utrecht University, Utrecht, The Netherlands

The scientific method has been characterised as consisting of two modes. On the one hand, there is the exploratory mode of science, where ideas are generated. On the other hand, one finds the confirmatory mode of science, where ideas are put to the test (Tukey, 1980; Jaeger & Halliday, 1998). Various alternative labellings of this apparent dichotomy exist: data-driven versus hypothesis-driven, hypothesis-generating versus hypothesis-testing, or night science versus day science (e.g., Kell & Oliver, 2004; Yanai & Lercher, 2020). Regardless of the labelling, the dichotomy of an “idea-generating” versus an “idea-testing” mode seems pervasive in scientific thinking.

The two modes of science appear to be differentially appreciated. For example, exploratory research may carry the stink of “merely a fishing expedition” (Kell & Oliver, 2004), or may be considered “weak” and yield unfavourable reviews (see the discussion of Platt [1964] in Jaeger & Halliday, 1998). Confirmatory research, on the other hand, seems to be considered as the holy grail in many areas of psychology (and vision science). Whether the appreciation for hypothesis-testing in psychology has been a reaction to the critique that theories in “soft areas of psychology” are “scientifically unimpressive and technologically worthless” (Meehl, 1978, p. 806) is an interesting question for debate. Nevertheless, the quintessential question in modern psychology is: “What is your hypothesis?” The correct answer one is expected to produce is a sentence at the level of a statistical analysis. Any other answer is wrong and yields the following response: “Ah, I see. You do exploratory research.” In Orwellian Newspeak “hypothesis” means “that which is to be decided on statistically” (cf. Yanai & Lercher, 2020), whereas “exploratory” means “descriptive” or even “unscientific.”

That the confirmatory mode of science is held in such high esteem is intriguing. *Confirmation* suggests that hypotheses or theories can be verified, a position diametrically opposed to that of, for example, Karl Popper, who claimed that theories can never be verified or confirmed, only refuted (e.g., Popper, 2002a). Note that this cuts right into the heart of discussions on whether science can be inductive and rational or not (Lakatos, 1978). It is not trivial semantics! But one does not find that a “refutatory” mode of science holds sway. Rather, refutation (or disconfirmation) is commonly avoided by the construction of ad-hoc auxiliary hypotheses when the data do not match with the theory (cf. the practice of Lakatosian defence, Meehl, 1990). Although sometimes frowned upon, ad-hoc hypotheses are not without merit. The observation of the planet Neptune by Galle in 1846 followed the ad-hoc hypothesis by Le Verrier and Adams (as discussed in Gershman, 2019): a great success for science. That ad-hoc hypotheses may also fail is evident from the hypothesised planet Vulcan by the same Le Verrier. That planet was never observed, although the discrepancy it addressed later proved to be relevant for Einstein’s theory of general relativity.

The discussion of confirmation versus refutation aside, the two-mode view of science is not merely a theoretical fancy that researchers debate about. It pervades increasingly more of the practicalities that researchers are faced with. The pre-registration movement, for example, seems to be built on this strict dichotomy. The Center for Open Science^1^ writes that “Preregistration separates hypothesis-generating (exploratory) from hypothesis-testing (confirmatory) research” and this “planning improves the quality and transparency of your research.” Note the explicit normative statement here. But is a strict dichotomy of exploratory research (or data-driven or hypothesis-free) versus confirmatory research (or hypothesis-testing) sensible at all?

Is there such a thing as hypothesis-free exploration? Consider the case of a person sitting in their yard, peering at a pond through binoculars. Can we claim that this person is observing the world without hypotheses? According to Popper (2002b), we cannot. He states: “observation is always observation in the light of theories” (p. 37). This need not be a formalised hypothesis according to the hypothetico-deductive method. Science is a human affair after all, it piggybacks on perception and cognition, which thrive through instinct, intuition, hunches, anticipation, quasi-informed guesses, and expertise (cf. Brunswik, 1955; Riegler, 2001; Chater et al., 2018). Such proto-hypotheses (Felin et al., 2021) do not always lend themselves neatly to verbalisation or formalisation, or are fuzzy at best (Rolfe, 1997). Thus, the decisions of where to sit, what direction to peer in, what binoculars to use, how long to wait are hardly hypothesis-free.

At the other extreme, one can ask whether hypothesis-testing is possible in the absence of exploration. Clearly, exploration in Tukey’s sense is crucial for forming a hypothesis in the first place: “Ideas come from previous exploration more often than from lightning strokes” (Tukey, 1980, p. 23). However, devising a critical experiment to put a hypothesis to the test inevitably involves exploration. Exploration of where in the stimulus-space to measure, which parameters to use for signal processing, and so forth. This should strike a chord with the experimental scientist. Theoretically, one might be able to conceive of an experiment that can be considered as purely “hypothesis-testing.” Yet, at best it would be the hypothetical limit on a continuum between the exploratory and confirmatory modes of science.

Thus, a strict two-mode view of science is too simplistic. Nevertheless, the practical implications of such a view may be substantial, also to those who abstain from initiatives such as pre-registration. In our experience, the strict two-mode view of science permeates the thinking of e.g., institutional review boards, ethics committees, and local data archiving initiatives. The procedures derived from this strict two-mode thinking tend to take on a Kafkaesque atmosphere: The bureau of confirmatory science will see you now. It will be most pleased to guide you on your way to doing proper science.

We are happy to concede that scientific studies may be characterised as being of a more or less exploratory nature and that some studies may be characterised as clear attempts to refute or decide between scientific hypotheses. We also understand that some procedures taken up by institutional review boards, ethics committees, journals (pre-registration), and so forth, are meant to counter phenomena such as “HARKing” (the evil twin of the ad-hoc auxiliary hypothesis), “p-hacking”, blatant fraud, or to increase the replicability of science (e.g., Nosek et al., 2012; Open Science Collaboration, 2015). Good intentions do not solely validate the means, however. What we vehemently oppose is the adoption and dogmatic use of a simplistic model of science and the scientific method that all research should adhere to. Dogma has no place in science, nor has it proved particularly effective throughout the history of science (Feyerabend, 2010).

In our view, the dogmatic two-mode view of science obscures a deeper discussion—that of the goal or purpose of science. According to the influential paper by the Open Science Collaboration (2015) it is “that ultimate goal: truth” (p. 7). This contrasts starkly with a quote from Linschoten (1978):

The statement that science seeks truth is meaningless. The word “truth” either means too much or too little. It has no scientifically relatable meaning, unless truth is equivalent to relevant knowledge. Knowledge is relevant when it allows us to explain, predict, and control phenomena (p. 390).^2^

If one considers hypotheses to be true or false, scientific findings to be true or false, and theories to be true or false, then a purely confirmatory way of thinking makes sense. All efforts to replicate—that is, to decide on which findings are *really* true—using the right statistical (Gigerenzer, 2018) or methodological rituals (Popper, 2002b) will inevitably bring one closer to that truth. If one is less concerned with truth, and more with predicting tomorrow, then the exploratory versus confirmatory dichotomy is not all that relevant. One would rather have meaningful discussions about generalisability (Yarkoni, 2020) or representativeness (Brunswik, 1955; Holleman et al., 2020). Anything that will yield a better prediction of tomorrow is useful, whether arrived at through Popper’s hypothetico-deductive methods, a hunch, a fishing trip, or counterinductively. According to Feyerabend (2010, p. 1), “Science is an essentially anarchic enterprise: theoretical anarchism is more humanitarian and more likely to encourage progress than its law-and-order alternatives.” Science needs no dogma.

## Notes

1. https://www.cos.io/initiatives/prereg, accessed 14 May 2021.

2. In the original Dutch: “De uitspraak dat wetenschap waarheid zoekt, is zinloos. Het woord ‘waarheid’ betekent te veel of te weinig. Het geeft geen wetenschappelijk verbindbare betekenis, tenzij waarheid gelijkluidend is met relevante kennis. Kennis is relevant wanneer ze ons in staat stelt verschijnselen te verklaren, te voorspellen, en te beheersen.”
